# Criterion validity of The International Physical Activity Questionnaire-Short Form (IPAQ-SF) for use in clinical practice in patients with osteoarthritis

**DOI:** 10.1186/s12891-021-04069-z

**Published:** 2021-02-27

**Authors:** Kenth Louis Joseph, Hanne Dagfinrud, Anne Christie, Kåre Birger Hagen, Anne Therese Tveter

**Affiliations:** 1 National Advisory Unit on Rehabilitation in Rheumatology, The Division of Rheumatology and Research, Diakonhjemmet Hospital, Oslo, Norway; 2 Faculty of Medicine, Institute of Health and Society, University of Oslo, Oslo, Norway; 3 Division of Health Service, Norwegian Institute of Public Health, Oslo, Norway

**Keywords:** Physical activity assessment, Criterion validity, IPAQ-SF, Accelerometer, Osteoarthritis, Clinical practice

## Abstract

**Background:**

To tailor physical activity treatment programs for patients with osteoarthritis, clinicians need valid and feasible measurement tools to evaluate habitual physical activity. The widely used International Physical Activity Questionnaire-Short Form (IPAQ-SF) is not previously validated in patients with osteoarthritis.

**Purpose:**

To assess the concurrent criterion validity of the IPAQ-SF in patients with osteoarthritis, using an accelerometer as a criterion-method.

**Method:**

Patients with osteoarthritis (*n* = 115) were recruited at The Division of Rheumatology and Research at Diakonhjemmet Hospital (Oslo, Norway). Physical activity was measured by patients wearing an accelerometer (ActiGraph wGT3X-BT) for seven consecutive days, followed by reporting their physical activity for the past 7 days using the IPAQ-SF. Comparison of proportions that fulfilled physical activity recommendations as measured by the two methods were tested by Pearson Chi-Square analysis. Differences in physical activity levels between the IPAQ-SF and the accelerometer were analyzed with Wilcoxon Signed-Rank Test and Spearman rank correlation test. Bland-Altman plots were used to visualize the concurrent criterion validity for total- and intensity-specific physical activity levels.

**Results:**

In total, 93 patients provided complete physical activity data, mean (SD) age was 65 (8.7) years, 87% were women. According to the IPAQ-SF, 57% of the patients fulfilled the minimum physical activity recommendations compared to 31% according to the accelerometer (*p* = 0.043). When comparing the IPAQ-SF to the accelerometer we found significant under-reporting of total physical activity MET-minutes (p = < 0.001), sitting (p = < 0.001) and walking (*p* < 0.001), and significant over-reporting of moderate-to-vigorous physical activity (p < 0.001). For the different physical activity levels, correlations between the IPAQ-SF and the accelerometer ranged from rho 0.106 to 0.462. The Bland-Altman plots indicated an increased divergence between the two methods with increasing time spent on moderate-to-vigorous intensity physical activity.

**Conclusion:**

Physical activity is a core treatment of osteoarthritis. Our finding that patients tend to over-report activity of higher intensity and under-report low-intensity activity and sitting-time is of clinical importance. We conclude that the concurrent criterion validity of the IPAQ-SF was weak in patients with osteoarthritis.

## Background

Tailored physical activity (PA) is the cornerstone in treatment of patients with osteoarthritis (OA) [1]. To curb disease specific symptoms like pain and joint stiffness as well as reduce the risk of cardiovascular co-morbidities [1–5], PA of moderate-to-vigorous intensity is found effective and is recommended throughout the course of the disease [1, 6–8]. To provide individually tailored PA treatment programs with optimal exercise dosage, clinicians need valid and feasible measures to evaluate their patients’ habitual PA level [9]. In clinical practice, the measurement tools must be easy to administer at a low cost and pose minimal patient burden, but still provide valid information.

PA is defined by requirement of increased energy expenditure [10]. PA energy expenditure in free-living environments can be assessed by the gold standard doubly labelled water method, by activity monitors, heart rate monitors, pedometers, diaries or by questionnaires [11]. The doubly labelled water method provides information solely on individuals’ energy expenditure [11, 12]. This method does not give information on habitual PA profile in terms of intensity, frequency or duration of the activity, which is important when evaluating patients’ PA levels [1]. However, activity monitors such as accelerometers, provide daily profiles on habitual PA [11, 13]. Accelerometers also allow for calculation of PA energy expenditure and weekly time in intensity specific PA (e.g. according to PA recommendations). Accelerometers are shown to agree well with the doubly labelled water method and may therefore serve as a criterion-method for measuring habitual PA [12, 14, 15].

Due to the lack of immediate information on PA from accelerometers, self-report questionnaires are often used in clinical practice. Questionnaires are easy to administer, have a low cost, and pose minimal burden on patients and clinicians, but a challenge is that they are prone to recall- and reporting bias [16, 17]. A questionnaire that is frequently used in research and clinical practice is The International Physical Activity Questionnaire-Short Form (IPAQ-SF) [18–21]. The IPAQ-SF was originally developed and validated for population surveys [22]. The validity of the questionnaire has been assessed in some disease-groups [19], but evidence on criterion validity in patients with OA is lacking [18, 20, 21]. Knowledge about criterion validity is important when assessing PA [20, 21], and in the recommendations for PA in people with OA and inflammatory arthritis the need for valid and feasible methods for assessing PA in clinical practice is underlined [1]. Thus, the purpose of the present study was to assess the concurrent criterion validity of the IPAQ-SF in patients with OA using an accelerometer as a criterion-method.

## Methods

### Subjects and design

Patients who attended a one-day OA patient education program at The Division of Rheumatology and Research at Diakonhjemmet Hospital (Oslo, Norway) were consecutively recruited from November 2017 to June 2018. Attendance at the program required a referral confirming an OA diagnosis from a doctor at the hospital a or by their general practitioner. Individuals ≥18 years of age, independent of walking aids and competent in verbal- and written Norwegian were eligible for inclusion. Exclusion criteria were patient’s incapable of ambulatory movement and/or the inability to understand verbal- and written Norwegian. Eligible patients were given verbal- and written information about the study, and those who provided written, informed consent were included. For excellent methodological quality, a minimum of 100 patients are recommended [23, 24]. To account for possible lack of compliance with using the accelerometer, a total of 115 patients were recruited.

Patients who consented to participate received a study pack including: the IPAQ-SF, an accelerometer, a diary on accelerometer wear-time, an instruction sheet on how to wear the accelerometer and a questionnaire on demographics.

Patients were instructed to wear the accelerometer mounted on the right hip (by an adjustable elastic belt) for all time awake except during water-based activities for seven consecutive days. On day six, a text message was sent to remind the patients to answer the questionnaires either on the seventh day before going to bed or in the morning on day eight, and to return the IPAQ-SF, the accelerometer, the wear-time diary, and the demographic questionnaire. in a pre-paid envelope.

### The IPAQ-SF (the international physical activity questionnaire - short form)

The previously translated Norwegian version of The IPAQ-SF (available at www.ipaq.ki.se) was used to assess self-reported PA [22]. The IPAQ-SF addresses the number of days and time spent on PA in moderate intensity, vigorous intensity and walking of at least 10-min duration the last 7 days, and also includes time spent sitting on weekdays the last 7 days [22]. The IPAQ-SF sum score is expressed in PA Metabolic Equivalent of Task (MET)-minutes per day or week. In the present study, data processing and analysis were calculated according to the official IPAQ scoring protocol (www.ipaq.ki.se). Total weekly PA MET-minutes were estimated by adding up the calculated MET-minutes within each PA intensity level (moderate intensity = 4.0 MET, vigorous intensity = 8.0 MET and walking = 3.3 MET). The reported time spent on sitting was calculated as time per weekday. In addition, reported time on moderate and vigorous physical activity (MVPA) were summed and expressed as MVPA-minutes per week and as MVPA MET-minutes per week. Finally, to categorize patients fulfilling or not fulfilling the PA recommendations, MVPA-minutes (≥150 or < 150 MVPA-minutes per week) and vigorous intensity PA (≥75 or < 75 min per week) were dichotomized [1].

### The accelerometer

The ActiGraph wGT3X-BT accelerometer (ActiGraph, LLC, Pensacola, FL) was used as a criterion measure of habitual PA. The accelerometer provides data on daily structure of habitual PA over 1–4 weeks, and is a frequently used and validated method [11, 12, 14, 15]. The tri-axial accelerometer continuously records ambulatory movement as counts per minute (CPM) were count-thresholds correspond to different PA intensity levels [13, 25]. Data were downloaded in 1-min time intervals obtained from the vertical axis using the associated licensed ActiLife software (version 6.13.3, ActiGraph, LLC). Valid PA registration was defined as minimum 4 days of at least 10 h of recording per day [25].

Total registration time was defined as 18 h of recording (from 6:00 a.m. to 00:00 a.m.) [26]. Non wear-time was defined as at least 60 consecutive minutes of zero counts (with allowance for ≤2 min with counts below 100), and wear-time was determined by subtracting non wear-time from 18 h [25]. Thresholds for different PA intensities were set to: ≥5999 CPM (vigorous); ≥2020–5998 CPM (moderate); 100–2019 CPM (light) and < 100 CPM (sedentary) [25].

The following calculations were done to make the accelerometer data comparable to the IPAQ-SF outcomes:

1. Total weekly time in different PA intensities was calculated by summing the recorded time within each threshold on valid days (in at least 10-min bouts, allowing for ≤2 min below the respective thresholds), then averaged over number of valid days, and finally multiplied by seven (1 week).
2. Total weekly PA MET-minutes were estimated by applying MET values congruent to the IPAQ-SF MET values, were the corresponding value for moderate intensity was 4.0 MET, vigorous intensity 8.0 MET and for light intensity 3.3 MET. Light activity (100–2019 CPM) was compared to IPAQ-SF walking. Sedentary time on valid weekdays was averaged over valid days and multiplied by 5 (weekdays).
3. Based on these variables, time on moderate and vigorous physical activity (MVPA) were summed and expressed as MVPA-minutes per week and MVPA MET-minutes per week.
4. Finally, to categorize patients fulfilling or not fulfilling the PA recommendations, MVPA-minutes (≥150 or < 150 MVPA-minutes per week) and vigorous intensity PA (≥75 or < 75 min per week) were dichotomized [1].

The diary on accelerometer wear-time (hours on and off each day), included questions concerning activities such as swimming, bicycling/ergometer-cycling, resistance exercise (using weights/apparatus) and cross-skiing/roller-skiing during the measurement week. The answer options on each activity were yes/no/don’t know/don’t remember (if yes; how many days and average time per day).

### Patients demographics

Demographic characteristics were collected by self-report, and comprised age (years), gender, height (cm), weight (kg), smoking habits (current, previous, never), educational level (primary school, upper secondary school, < 4 years college/university, ≥4 years college/university), currently working (yes/no, if no; student, retired, well-fare, sick leave, else), civil status (living alone, living with someone), pain during the last week (Numeric Rating Scale (NRS) ranging from ‘0’ no pain to ‘10’ worst imaginable pain) and general health (EQ-5D visual analog scale ranging from ‘0’ worst imaginable health to ‘100’ best imaginable health). Specific OA joint was not recorded.

## Statistics

Data are presented as frequency (n) and proportion (%), mean and standard deviation (SD) or median and interquartile range (IQR, 25th and 75th percentile). Body Mass Index (BMI) was calculated by weight and height (kg/m^2^) and was categorized into normal weight (BMI < 25), overweight (BMI ≥25 to < 30) and obese (BMI ≥30). Data on education level were dichotomized into ≥1 year or < 1 year of college/university. Those reporting ‘yes’ or ‘no’ on currently working were categorized as ‘employed’ and ‘not employed’, respectively (reporting ‘yes’ on currently working in combination with ‘retired’, ‘sick leave’ or ‘else’ was categorized as ‘employed’). Pain was defined as none to mild pain (NRS ≤5) and moderate to severe pain (NRS ≥6).

In our analyses, we included those with valid accelerometer data and complete data on moderate and vigorous PA by the IPAQ-SF. The difference between the corresponding IPAQ-SF and accelerometer measured PA levels were tested with Wilcoxon Signed-Rank Test. The mean differences and 95% confidence interval (95% CI) that were calculated by independent t-test are shown. Proportions fulfilling the PA recommendations according to the two methods were compared by Pearson Chi-Square analysis.

Concurrent criterion validity of the IPAQ-SF against the corresponding accelerometer measured PA levels was analyzed by Spearman rank test. Correlation coefficients (rho) of ≤0.10 were defined as negligible, 0.10–0.39 as weak, 0.40 to 0.69 as moderate and ≥ 0.70 as strong/very strong [27]. Based on previous studies, we hypothesized weak to moderate correlations between the different PA levels measured by IPAQ-SF and the accelerometer [28, 29]. The mean difference in measured PA levels between the two methods was also visualized by Bland-Altman plots with 95% limits of agreement [30].

Due to the accelerometers’ limitations on recording activities such as bicycling, resistance exercises, skiing and swimming (the accelerometer is taken off during water-based activities), we examined if the discrepancy between the two methods in weekly MVPA-minutes were different between patients reporting and not reporting such activities using Mann-Whitney U test. Significance levels were set to *P* < 0.05 in all analyses. Statistical analyses were calculated using IBM SPSS Statistics Version 21.

## Results

**Fig. 1:**
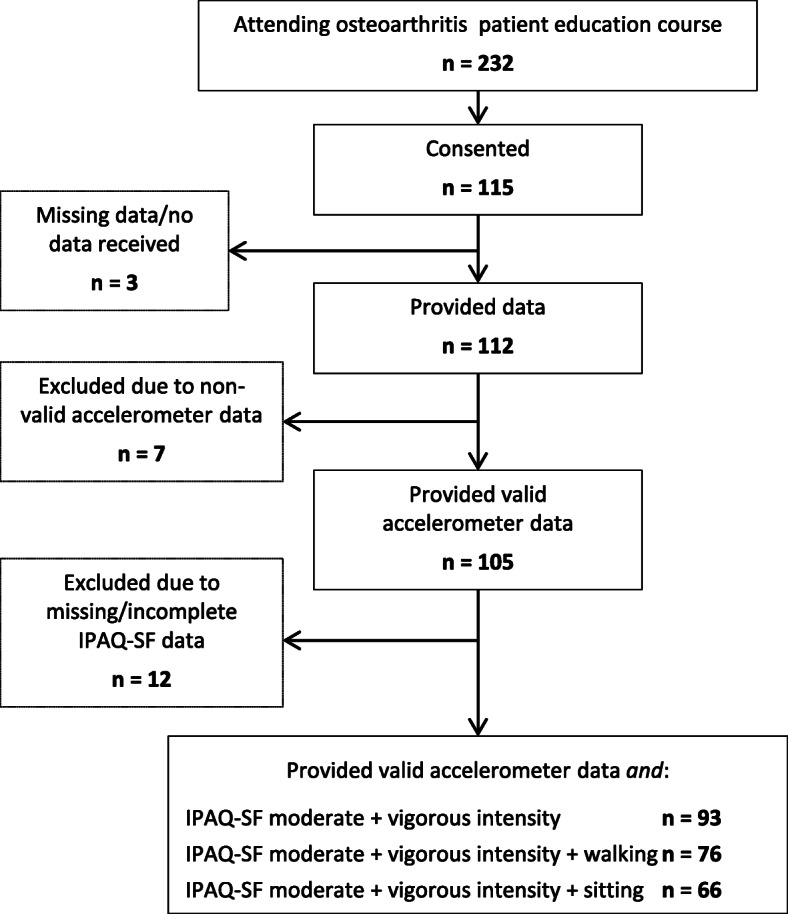
Flowchart of patients that provided data and were included in the analyses

In total, 105 patients returned valid accelerometer data (Fig. 1), and 96% had five or more valid days with ≥10 h of recording per day. Mean (SD) wear-time was 13.9 (1.2) hours per day. Among patients with valid accelerometer data, 93 (89%) provided IPAQ-SF-data on both moderate and vigorous PA level (Fig. 1). The reasons for missing/incomplete PA data were mostly missing response and/or a ‘don’t know’ response on moderate and/or vigorous intensity in the IPAQ-SF. No statistical differences were found in demographic characteristics between the 93 patients that were included for analyses and the 19 patients that had missing/incomplete PA data (data not shown).

Demographic characteristics are shown in Table 1. Half of the patients (49%) were categorized as overweight/obese (BMI ≥25). Almost one in three (28%) scored moderate to severe pain (NRS ≥6).

**Table 1:** Demographic characteristics of patients with osteoarthritis, *n* = 93

Age (years), mean (SD)	64.8 (8.7)
Women, n (%)	81 (87.1)
BMI (kg/m2), mean (SD)^a^	25.5 (3.9)
Normal weight (BMI < 25), n (%)	46 (51.1)
Overweight (BMI ≥25 to < 30), n (%)	32 (35.6)
Obese (BMI ≥30), n (%)	12 (13.3)
Smoking, n (%)	
Current	8 (8.6)
Previous	49 (52.7)
Never	36 (38.7)
Education level ≥ 1 year college/university, n (%)	63 (67.7)
Employed, n (%)^b^	42 (46.2)
Not employed status, n (%)	
Retired	37 (40.6)
Well-fare/sick leave/else	12 (13.2)
Civil status, living alone	36 (38.7)
Pain (NRS, 0–10), mean (SD)^c^	4.3 (2.1)
General health (EQ-5D-VAS, 0–100), mean (SD)^b^	64.3 (18.8)

*BMI* Body mass index; *NRS* Numeric rating scale; *EQ-5D-VAS* EuroQol-5D-Visual Analog Scale; *IQR* Interquartile range.

^a^, *n* = 90 due to missing height/weight data to calculate BMI

^b^, *n* = 91 due to missing data

^c^, *n* = 92 due to missing data

More than half of the patients (57.0%) fulfilled the PA recommendations (of minimum 150 min of at least moderate PA per week) according to IPAQ-data, while one in three (31.2%) fulfilled the recommendations according to accelerometer data (Table 2).

**Table 2:** Physical activity values assessed by IPAQ-SF and accelerometer, including *p*-values for differences (*n* = 93)

	IPAQ-SF	Accelerometer	Mean difference (95% CI)	Difference ***p***-value
PA guidelines^*^, n (%)				
Proportion fulfilling PA guidelines	53 (57.0)	29 (31.2)		0.043^c^
*PA levels, median (IQR)*				
Total PA MET-minutes, per week	1985 (898, 4217) ^a^	4059 (2712, 5467)	− 1616 (− 2096, − 1137)	< 0.001 ^d^
MVPA MET-minutes, per week	780 (120,1680)	238 (45, 648)	775 (445, 1104)	< 0.001 ^d^
MVPA-minutes, per week	180 (30, 300)	60 (11, 162)	118 (53, 183)	< 0.001 ^d^
Vigorous PA, minutes per week	15 (0, 120)	0 (0, 0)	70 (45, 96)	- ^e^
Moderate PA, minutes per week	90 (0, 210)	60 (11, 162)	53 (−1, 107)	0.272 ^d^
Walking/light PA, minutes per week	245 (105, 630) ^a^	1101 (711, 1475)	− 729 (− 865, − 593)	< 0.001 ^d^
Sitting/sedentary time, hours per weekday	6.0 (4.0, 10.0) ^b^	8.9 (7.8, 10.0)	−1.8 (−2.5, − 1.1)	< 0.001 ^d^

95% CI, 95% confidence interval; *IPAQ-SF* The International Physical Activity Questionnaire-Short Form; *MVPA* moderate to vigorous intensity physical activity; *MET* Metabolic Equivalent of Task.

Data given in frequency (percent) for ‘Proportion fulfilling PA guidelines’ and median (interquartile range, IQR) for ‘PA levels’.

*Fulfilling PA guidelines, ≥150 min of MVPA or ≥ 75 min of vigorous PA per week

**Fig. 2:**
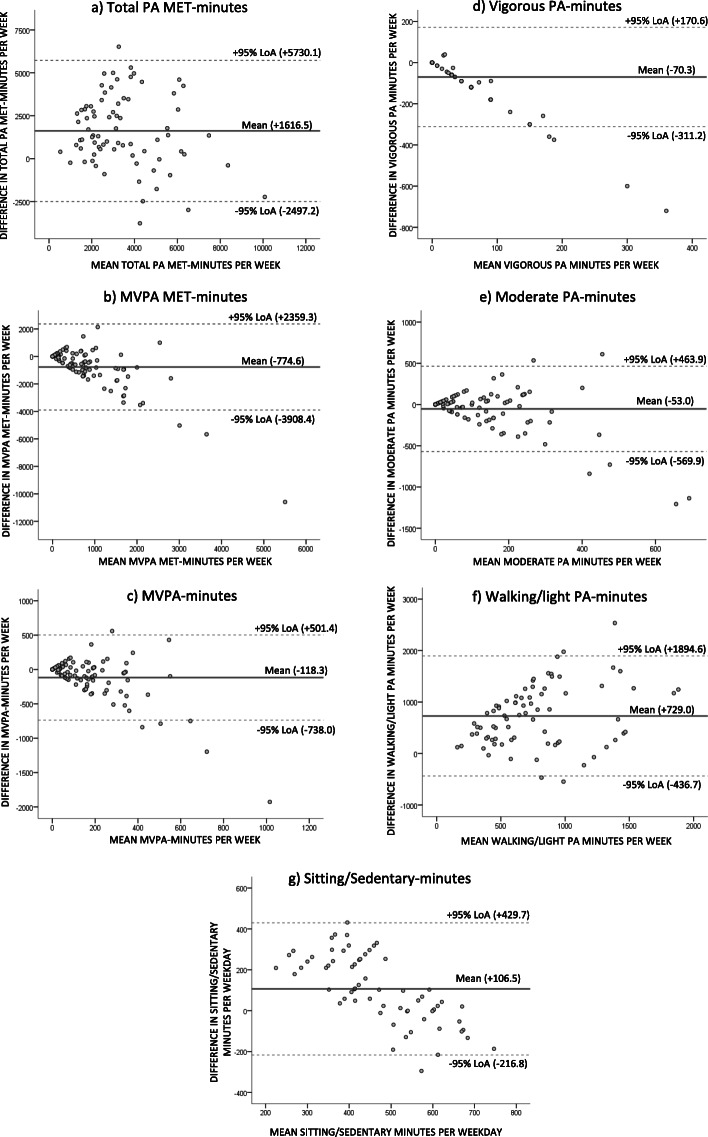
Bland-Altman plots of the agreement between self-reported PA (IPAQ-SF) and accelerometer measured PA illustrated with mean difference and 95% Limits of Agreement (LoA)

When comparing PA levels measured with the two methods, significant differences were found for total PA MET-minutes and all intensity specific PA levels (*p* < 0.001), except moderate intensity (*p* = 0.272). The patients reported lower total PA MET-minutes, less time sitting and walking, and more time in moderate and vigorous PA compared to what was measured by the accelerometer (Table 2). The correlation between PA levels from the IPAQ-SF and the corresponding PA levels from the accelerometer ranged from rho 0.106 to 0.462, were sitting time correlated moderately, and total PA MET-minutes and all intensity specific PA levels correlated weakly (Table 3). The Bland-Altman plots visualized that the difference between the methods increased with increasing minutes of PA for moderate intensity, MVPA-minutes and MVPA MET-minutes (Fig. 2, b, c, e).

**Table 3:** Spearman correlations (rho) with *p*-values between IPAQ-SF and accelerometer measured PA levels, *n* = 93

*PA levels*	Correlation	*p*-value
Total PA MET-minutes, per week ^a^	0.373	0.001
MVPA MET-minutes, per week	0.315	0.002
MVPA-minutes, per week	0.329	0.001
Vigorous PA, minutes per week	0.106	0.311
Moderate PA, minutes per week	0.275	0.008
Walking/light PA, minutes per week ^a^	0.145	0.210
Sitting/sedentary time, hours per weekday ^b^	0.462	< 0.001

Finally, 59 patients reported to spend a median (IQR) of 120 (60, 222) minutes on one or more activities inadequately captured by the accelerometer (cycling, resistance exercises, skiing or swimming). However, for the difference between IPAQ-SF and accelerometer measured time spent on MVPA, no statistical between-group difference was found (*p* = 0.560).

^a^, *n* = 76 due to incomplete response on IPAQ-SF.

^b^, *n* = 66 due to in incomplete response on IPAQ-SF.

^c^, *p*-value for difference calculated by Pearson Chi-Square analysis.

^d^, p-value for difference calculated by two-tailed paired Wilcoxon Signed Ranks Test.

^e^, p-value by Wilcoxon analysis not included because calculations resulted in 44 ties with zero vigorous minutes between the IPAQ-SF and the accelerometer.

^a^, *n* = 76 due to incomplete response on IPAQ-SF.

^b^, *n* = 66 due to incomplete response on IPAQ-SF.

## Discussion

In the present study, we compared self-reported physical activity (IPAQ-SF) with concurrent, objectively measured activity (accelerometer) in patients with OA. The main findings were that the patients overestimated self-reported moderate and vigorous activity and underestimated light activity, sitting time and total PA MET-minutes compared to data obtained with the accelerometer.

Based on self-reported PA, we found that more than half of the patients fulfilled the PA recommendations, but only one third of the patients were sufficiently active according to data from the accelerometer. In clinical practice it is important to identify patients that do not fulfill the PA recommendations. However, as our results indicate, these patients may not necessarily be identified by self-reporting their physical activity, which may result in sub-optimal disease management and increased risk of comorbidity.

In our study, we found weak correlations between self-reported and objectively measured total PA MET minutes and the different PA levels. This is in line with results from studies in individuals with self-reported OA [29], hip OA [31], hip- or knee arthroplasty [28] and in the general population [26], showing similar weak to moderate correlations between various self-reported and objectively measured PA levels. Our results underline that PA measures from the two methods cannot be used interchangeably. Important findings of our study were also that participants’ under-reported total PA MET-minutes and time spent on light activities, while they reported three times more MVPA-minutes than recorded by the accelerometer. Further, this discrepancy increased with more self-reported time in MVPA. Divergence between the methods can be explained by recall- and reporting bias related to use of questionnaires [16]. For example, walking is an everyday, “unconscious” activity that can be difficult to report in detail, whereas the accelerometer records walking with high accuracy [32]. The underreporting of time spent on walking may indicate that people pay less attention to light, every-day activity than to more intensive activity. Another possible explanation may be that walking is experienced as strenuous in patients with pain and stiffness due to hip- and knee OA, and therefore reported as more vigorous than shown by accelerometer. Since walking is under-reported and MVPA over-reported, the results in our study underline that recording solely total PA minutes is not sufficient when evaluating patients’ habitual PA.

Pain is a predominant symptom in OA [2], and patients may therefore prefer to engage in moderate to vigorous activities that cause less joint-related pain, i.e. resistance exercises, bicycling or swimming. However, such activities are inadequately recorded by a hip-worn accelerometer. We hypothesized that the type of activity could explain some of the discrepancy between the two methods in our study, but statistical analyses rejected this hypothesis, as no difference in over-reporting of MVPA-minutes was found between those engaging and not engaging in such activities.

In the present study, we found that 57% of the patients fulfilled the PA recommendations based on data from the IPAQ-SF, whereas only 31% was sufficiently active based on data from the accelerometer. Similar results are shown in a population-based OA study, in which half of the patients reported PA levels meeting the recommendations compared to only < 15% according to data from accelerometers [29]. Clinicians must be aware that even if patients report adequate PA, they may not really meet the PA recommendations. Sufficiently dosed PA is shown to be an effective treatment alternative for patients with musculoskeletal diseases, leading to less pain, improved physical function [6–8] and improved cardiorespiratory fitness which in turn is associated with reduced risk of cardiovascular disease [1, 33]. Thus, identifying patients not fulfilling the PA recommendations is important to provide optimal disease management for this large group of patients.

Our study has some limitations. A potential selection bias may be present as patients attending an educational course probably have a more positive attitude towards and are more likely to adhere to treatment recommendations. Additionally, most of the included patients were women, and the validity of the results for men with OA is therefore unclear. However, the majority of patients with OA are women [29, 34, 35], and the results may thereby be valid for the population seen in clinical practice. Further, we targeted a sample of 100 patients and 93 patient provided sufficient PA data. In rating of methodological quality of studies on measurement properties it is suggested that samples of 50 is good and 100 is excellent [24]. In our study we analyzed a sample close to 100, and we found no differences in any demographic characteristics between those with and without sufficient PA data. Lastly, the accelerometer used as a criterion-method is not validated in patients with OA [36]. Considering that resting metabolic rate, gender, BMI, and work economy are shown to affect the accuracy of accelerometer measured PA [37, 38], combining accelerometry with heart rate monitoring may improve the accuracy in estimating energy expenditure of habitual PA [39, 40]. Future studies should investigate the accelerometers accuracy in classifying PA intensity in patients with OA, including different subgroups (i.e. OA phenotypes, gender, BMI, age). This is important knowledge in the search for optimal PA dosages in the treatment of OA. But, in lack of a true gold standard to measure habitual PA, accelerometry may be considered the best available single method.

In our study, we found that the patients reported a three-fold more time in high-intensity PA than what accelerometer recordings showed for the same days. An explanation for this could be that experienced intensity may be inflated due to OA symptoms like pain, fatigue or functional limitations. It is previously shown that responses on OA-specific questionnaires are strongly correlated with patients’ pain-level, while performance based tests are less influenced by pain [41, 42]. Accordingly, self-report and objective measures of PA do not necessarily provide similar, but rather complementary information, and both methods are needed to better understand the performance and experience of activity in patients with OA. Even if the accelerometer gives more accurate data on habitual activity, the self-report method may capture the patient’s experience of being active. This is valuable information for the clinician in helping patients to overcome barriers and motivate for activity as part of the disease management.

## Conclusion

We found that correlations between the objective criterion-method and the self-reporting in IPAQ-SF were weak. However, self-reporting PA may capture the patients’ experienced intensity of physical activity, which is important for clinicians in providing an optimal PA treatment program. Physical activity dosed according to guidelines is the most important treatment of OA, and the finding that patients with OA tend to over-report activity of higher intensity and under-report low-intensity activity and sitting-time is therefore of clinical importance.

## Data Availability

The datasets used and analysed during the current study are available from the corresponding author on reasonable request.

## Abbreviations

BMI: Body Mass Index
CI: Confidence Interval
CPM: Counts Per Minute
IPAQ-SF: International Physical Activity Questionnaire-Short Form
IQR: Interquartile Range
MET: Metabolic Equivalent of Task
MVPA: Moderate to Vigorous intensity Physical Activity
NRS: Numeric Rating Scale
OA: Osteoarthritis
PA: Physical Activity
SD: Standard Deviation
